# Optical microscopy and confocal data of aerosol jet printed lines over a 16-hour print duration

**DOI:** 10.1016/j.dib.2022.108080

**Published:** 2022-03-24

**Authors:** David Yoo, Clare M. Mahoney, James R. Deneault, Christopher Grabowski, Drake Austin, J. Daniel Berrigan, Nicholas Glavin, Philip R. Buskohl

**Affiliations:** a UES, Inc., Dayton, OH 45432; b Arctos, Dayton, OH 45432; c Air Force Research Laboratory, Wright-Patterson AFB, 45433

## Abstract

Optical microscopy images and confocal data for Aerosol Jet Printed (AJP) lines over a 16 hour print duration is provide in this dataset (“Mapping Drift in Morphology and Electrical Performance in Aerosol Jet Printing” [1]). Lines were uninterruptedly printed by AJP on a glass substrate using silver nanoparticle ink over a 16-hour time frame. The ink used for this experiment was a 0.6:0.3:0.2 mL mixture of Clariant Prelect TPS 50 G2 silver nanoparticle ink, ethylene glycol, and deionized water, respectively. Deposition was achieved with an Optomec AJ 300-UP Aerosol Jet^TM^ Deposition System using a Sprint Series Ultrasonic Atomizer MAX, aerodynamic filtering, and a nozzle having an orifice diameter of 150 µm. The typical focus ratio of 1.75 within standard range was used. The optical microscopic images of 350 µm AJP printed lines at 80 different time points were then selectively collected. Keyence VK-X200 with 150x magnification was used, which provided 50 µm to 267 pixel resolution image with more than 1000 cross-sections at each time point. Filtering of the pixels with outlying heights was performed with a multi-file analyzer. The dataset was primarily collected to understand system-level, temporal drifts in print morphology, which would further allow to predict electrical performance in time domain. Additional purposes for the dataset include: 1) benchmark dataset for morphology and print performance between AJP systems and print settings, 2) test data for new image filtering, segmentation, and classification algorithms and 3) baseline training data for real-time, *in situ* classification of operational time windows for AJP feedback control.

## Specifications Table

**Table:** 

Subject	Industrial and manufacturing Engineering, Electrical and electronic Engineering, Computational mechanics
Specific subject area	Aerosol Jet Printing, Morphology
Type of data	Image and Spreadsheet
How data were acquired	Microscope Images: • Keyence VK-X200 optical microscope with 150x magnification Confocal Data: • Keyence VK-X200 optical microscope with 150x magnification • Z-resolution: >100 µm depth with minimum step size of 2 µm • Keyence multi-file analysis: • Reference planes were set on substrate on both sides of line for tiltcorrection. • Pixels with strong variations in height compared to neighboring pixels were filtered.
Data format	Microscope Images: • Pixel format: RGB format (0-255,0-255,0-255) • Resolution of Image: 4562 by 1860 pixels • Spatial Calibration Factor: 0.187 µm/pixel (50 µm/267 pixels) Confocal Data: • Pixel format: Greyscale (Intensity: 0-1) • Resolution of Image: 400 by 1860 pixels • Spatial Calibration Factor: 0.187 µm/pixel • Intensity Range: 0 – 1, Height Range: 0 – 10 µm
Parameters for data collection	Room temperature, Atmospheric environment
Description of data collection	• Optical microscopy of aerosol jet printed Ag nanoparticle ink over a 16-hour print duration • Collected data: Microscopic images of 350 µm at 80 different time points
Data source location	Air Force Research Laboratory, Materials & Manufacturing Directorate Wright-Patterson AFB, Dayton, OH, United States of America
Data accessibility	Data is hosted with this Data-in-Brief article.
Related research article	Yoo, D., Mahoney, C.M., Deneault, J.R., Grabowski, C., Austin, D., Berrigan, J.D., Glavin, N., Buskohl, P.R. Mapping drift in morphology and electrical performance in aerosol jet printing. *Progress in Additive Manufacturing* (2021). https://doi.org/10.1007/s40964-021-00165-7

## Value of the Data

• The optical and profilometry data of aerosol jet printed lines over a 16-hour print time included in the dataset is useful for 1) understanding system-level, temporal drifts in print morphology and 2) benchmarking morphology and print performance between AJP systems and print settings over a 16-hour print time.
• The data can be used to study system-level transitions in print morphology of aerosol-based printing methods, serve as benchmark data to compare with endurance printing performance between printers and print settings, and serve as a benchmark dataset to compare line quality metrics over extended print times.
• The data may also serve as a useful test dataset for image filtering, segmentation, and classification algorithms, with specific interest for real-time print classification for automated AJP feedback control.
• The 3-dimensional profilometry data could potentially be used to study transitions in the cross-sections of AJP printed lines over 16 hour print times and serve as a benchmark dataset to compare with line cross-section optimization studies.
• Material science, additive manufacturing and image analysis researchers are the anticipated target audience to directly benefit from this dataset.

## 1 Data Description

**Fig. 1:**
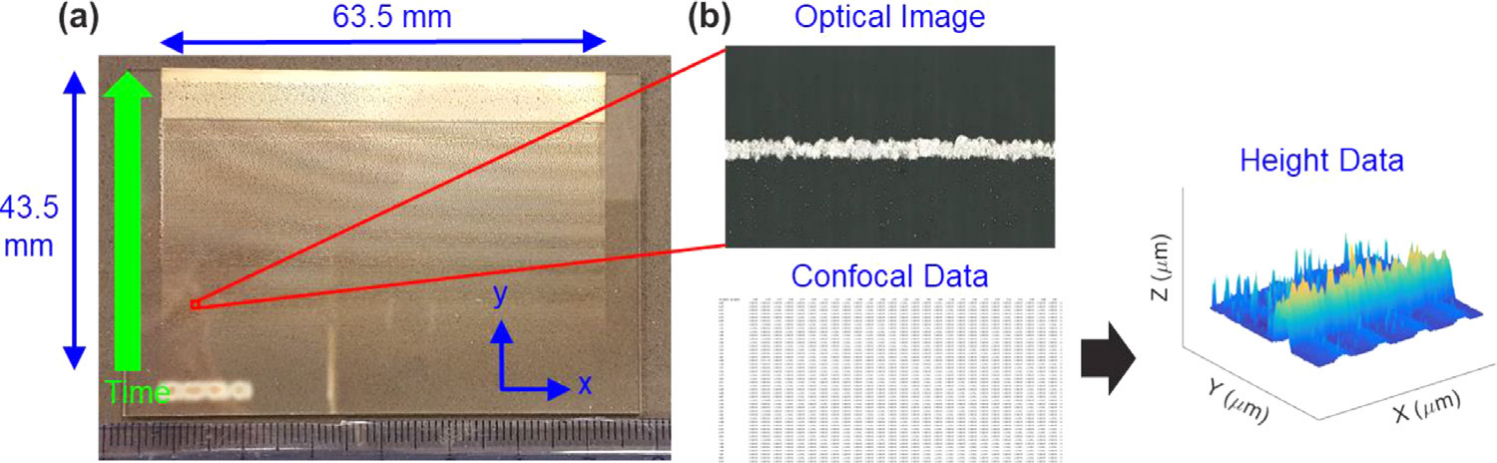
Schematic of location, time points and orientation of collected data. **a)** Global view of printed substrate with annotations for the regions of interest (ROI) included in this dataset. **b)** Representative ROI with spatial orientation and intensity calibration indicated, as included in the datafile.

Optical images and confocal profilometry data of AJP printed 350 µm line segments at select times over 16 hour print are included in this dataset. Fig. 1 (a) outlines the location, orientiation and print time of the data included in this study. Optical images and confocal data at each print time are obtained through Keyence microscope, as shown in Fig. 1 (b). Representative images of the original optical images and the corresponding confocal data can be both found in “Data in Brief – Drift_AJP_Manuscript.” folder. The optical images are saved as “Optical_Image_*xx*_*yy*.png” where *xx* and *yy* are starting and ending print times, respectively. The corresponding confocal data at print times of *zz* are saved as “*zz*” in spreadsheets in “Confocal_Data.xlsx”.

## 2 Experimental Design, Materials and Methods

### 2.1 AJP preparation and processing details

Deposition was achieved with an Optomec AJ 300-UP Aerosol Jet^TM^ Deposition System using a Sprint Series Ultrasonic Atomizer MAX, aerodynamic filtering, and a nozzle having an orifice diameter of 150 µm. The aerosol jet system's enclosure has been fitted with an external dehumidifier to keep the relative humidity of the print environment at a minimum (10-11% typ.) The ink used for this experiment was a 0.6:0.3:0.2 mL mixture of Clariant Prelect TPS 50 G2 silver nanoparticle ink, ethylene glycol, and deionized water, respectively. The specific ink composition was used in this study to provide good conductivity at 300,000 S/cm, and decent printability using ultrasonic atomization that narrow line widths are achievable (6-8 µm) with minimal overspray. A typical volumetric deposition rate for the silver ink we used in the study is 0.0000105 mL / minute (300 µm nozzle, atomizer gas flow rate (AGFR) = 36 sccm). At this rate, the 1.1 mL aliquot of ink is more than sufficient for this 16 hour drift study. Immediately prior to use, the glass substrates (Corning® water white glass, 75 mm × 50 mm × 1 mm) were hand-washed with Micro-90® industrial cleaning solution, rinsed with distilled water, and air plasma cleaned at 18W, 100 mTorr for 10 minutes with a Harrick PDC-32G Plasma Cleaner. After deposition, samples were sintered in air at 185 °C (ramp rate of 5.5 °C/min, hold for 60 minutes) using a Memmert UF 30 Plus programmable convection oven. The measured conductivity of the ink after the sintering was 300,000 S/cm. The surface tension and the viscosity of the ink were 35 ± 3 mN/m and 25 ± 2 cP, respectively. Optical micrographs and height data were collected using a Keyence VK-X200 with a z height resolution of approximately 40 nm.

For long-term drift analysis of line morphology, a 1.1 mL aliquot of diluted ink was prepared (as describe above) and a clean 400 mm length of 1/8” (OD) x 3/32” (ID) polypropylene tubing was installed to carry the aerosol to the 150 µm nozzle. This larger inner diameter tubing helps to mitigate the detrimental effects of the ink building up within the tube. We also employ a passive aerosol filter (US20190314849A1) immediately upstream of the nozzle assembly that redirects any ink drops that might form, run down the walls of the mist tube, and otherwise clog the nozzle. All other components were cleaned as appropriate by ultra-sonication in a solution of Micro-90® detergent and water followed by distilled water rinsing. The sheath and carrier gas flow rates were fixed at 52.5 sccm and 30 sccm, respectively, for the duration of the experiment. Straight 65 mm lines were printed at 0.225 mm intervals at 1.0 mm/s with a 200 s static deposition step between the printings of each line yielding a total line-to-line print time of 272.2 s. The platen was held at a constant 50 °C, the ultrasonic bath at 20.4 °C, and the ultrasonic atomizer current was set to maximum (593 mA), which becomes highly stable with more print duration and whose maximum variation throughout the experiment was less than 1.0%. The frequency of the ultrasonic atomizer was 1.7 MHz. The ambient conditions of the enclosure were 34.1 °C with a relative humidity of 11%. The specific drift tests included in this manuscripts were repeated twice.

### 2.2 Optical microscopy and confocal profilometry details

**Fig. 2:**
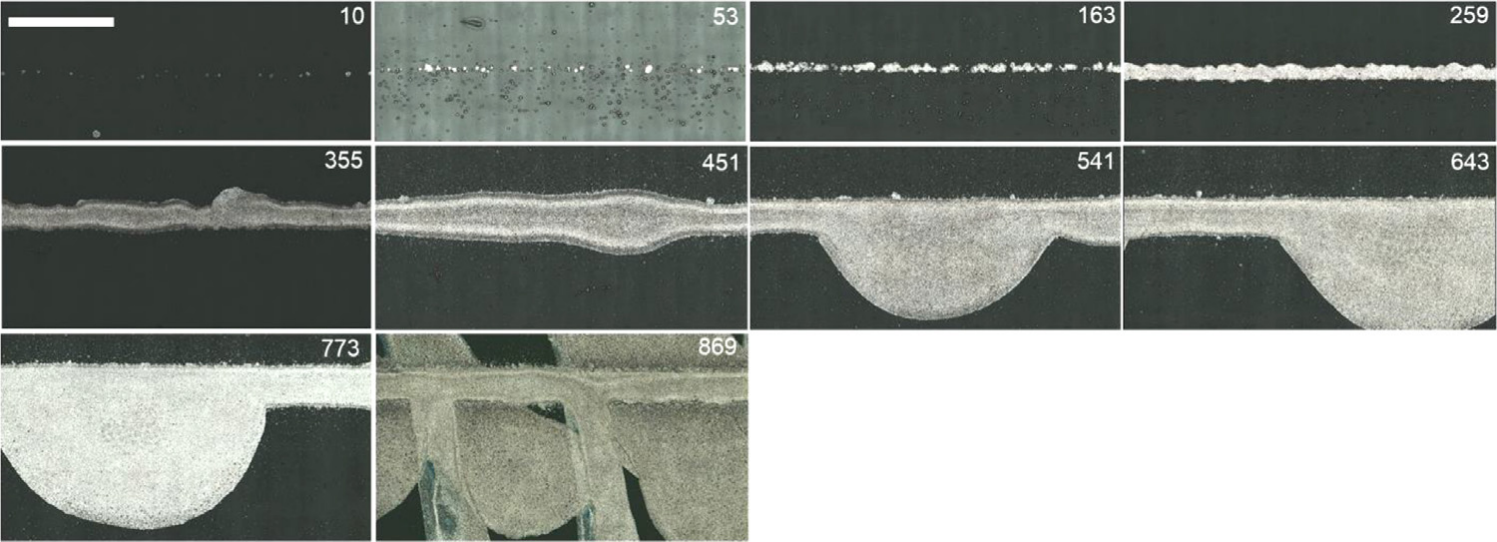
**Representative images of AJP print morphology across the 16-hour print.** The optical microscopic images of AJP printed 350 µm lines over print duration from 0 to 16 hours are shown. The scale bar represents 100 µm and upper right corner indicates approximate timepoints in minutes.

Optical micrographs and height data were collected using a Keyence VK-X200, and verified using a Bruker Dektak XT stylus profilometer. The optical microscopic images and confocal data of 350 µm lines at 80 different time points were obtained by Keyence VK-X200 with 150x magnification lens, which provided 50 µm to 267 pixel resolution image (see Fig. 2 for location and orientation details). The z height resolution of the confocal images was approximately 40 nm. Neutral density (ND) filter was applied to correct overexposure prior to scanning, and auto gain control function was used to further adjust intensity of light. Keyence multi-file analysis application was used to filter out pixels with outlying height data after scanning, for which pixels with strong variations in height compared to neighboring pixels were filtered. “Medium” was applied for the data. Reference planes were set on substrate on both sides of line for tilt correction. Bright and Dark Cut Levels (BCL, DCL) function was utilized to cut saturated pixels, and replace them using interpolation. The data were obtained in greyscale (intensity: 0-1), which were then precisely calibrated into height range of 0 – 10 µm.

## Declaration of Competing Interest

The authors declare that they have no known competing financial interests or personal relationships which have, or could be perceived to have, influenced the work reported in this article.

## Appendix Supplementary materials

Image, application 1

Image, application 2
