# Supplementary figures and images for: Optical microscopy and confocal data of aerosol jet printed lines over a 16-hour print duration
Source: Data Brief. 2022 Mar 24;42:108080. doi: 10.1016/j.dib.2022.108080 (PMC9010625; doi:10.1016/j.dib.2022.108080)

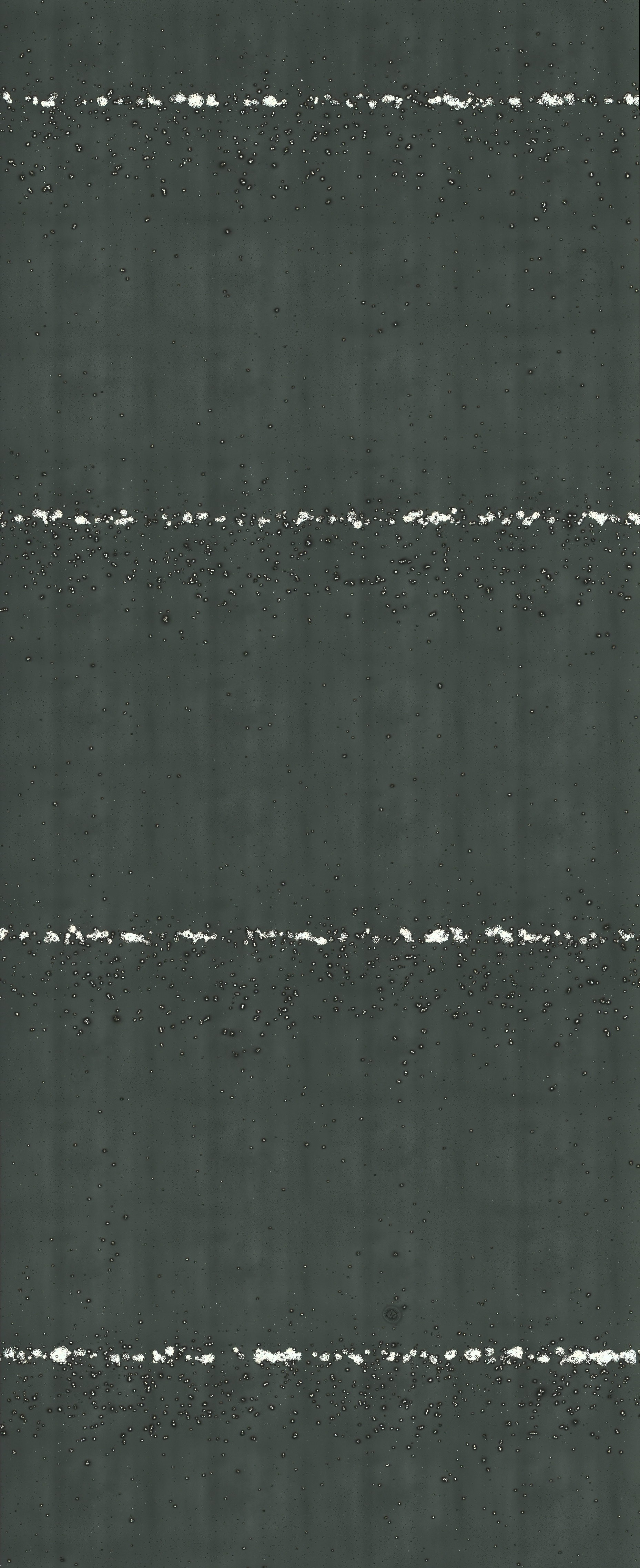

Supplement: Supplementary file 1 [file mmc1.zip › Optical_Image_101_to_115_mins.png]

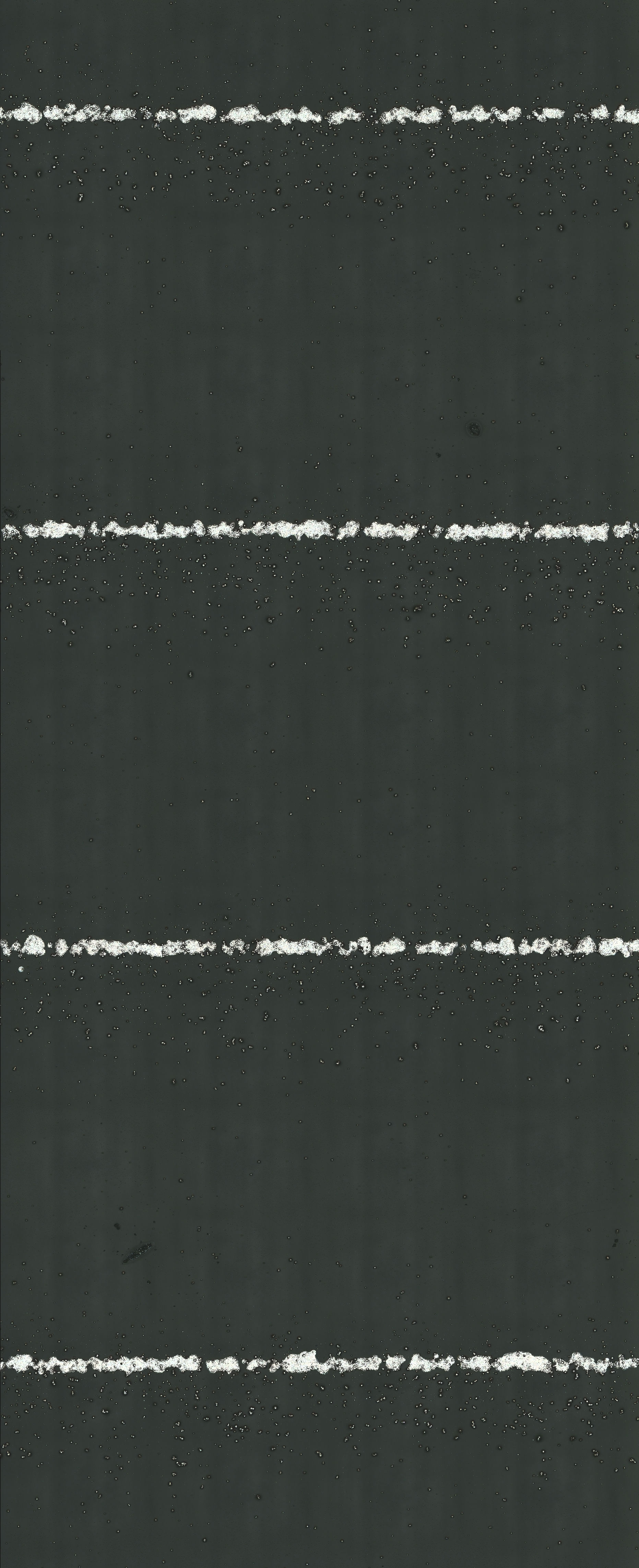

Supplement: Supplementary file 1 [file mmc1.zip › Optical_Image_149_to_163_mins.png]

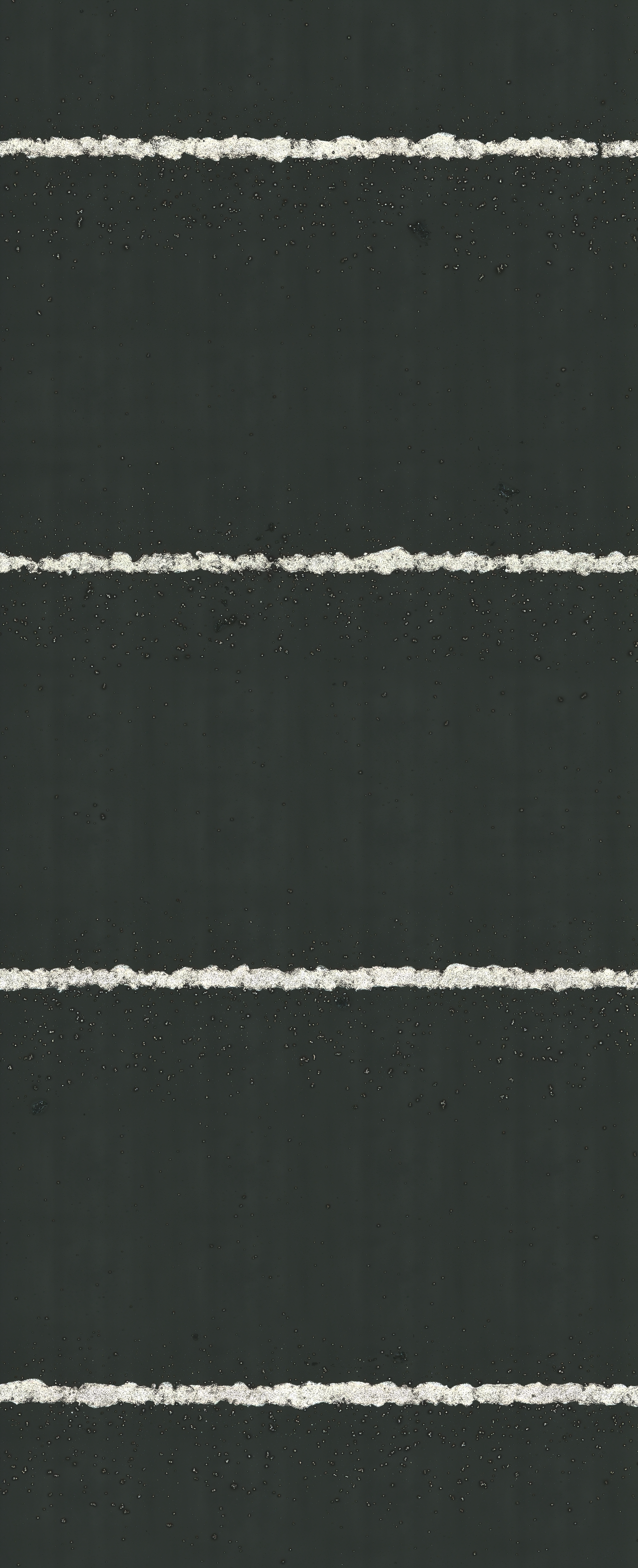

Supplement: Supplementary file 1 [file mmc1.zip › Optical_Image_197_to_211_mins.png]

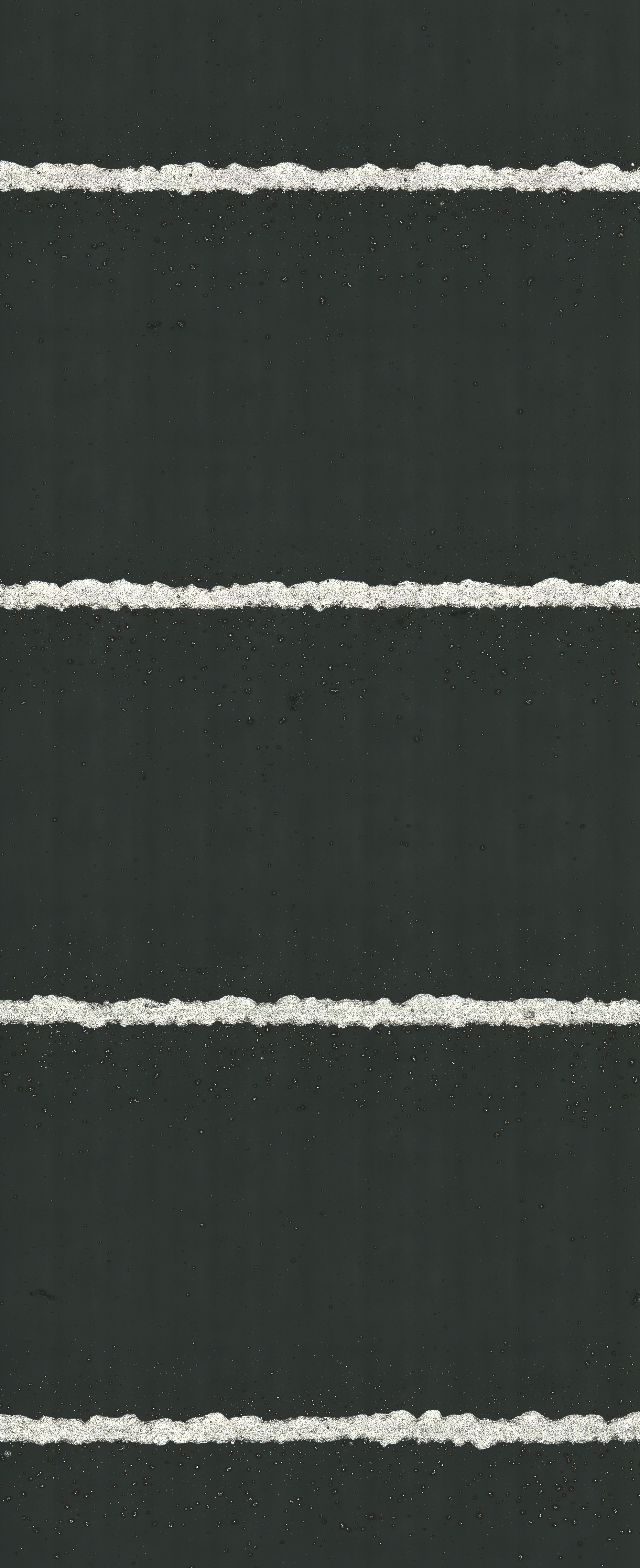

Supplement: Supplementary file 1 [file mmc1.zip › Optical_Image_245_to_259_mins.png]

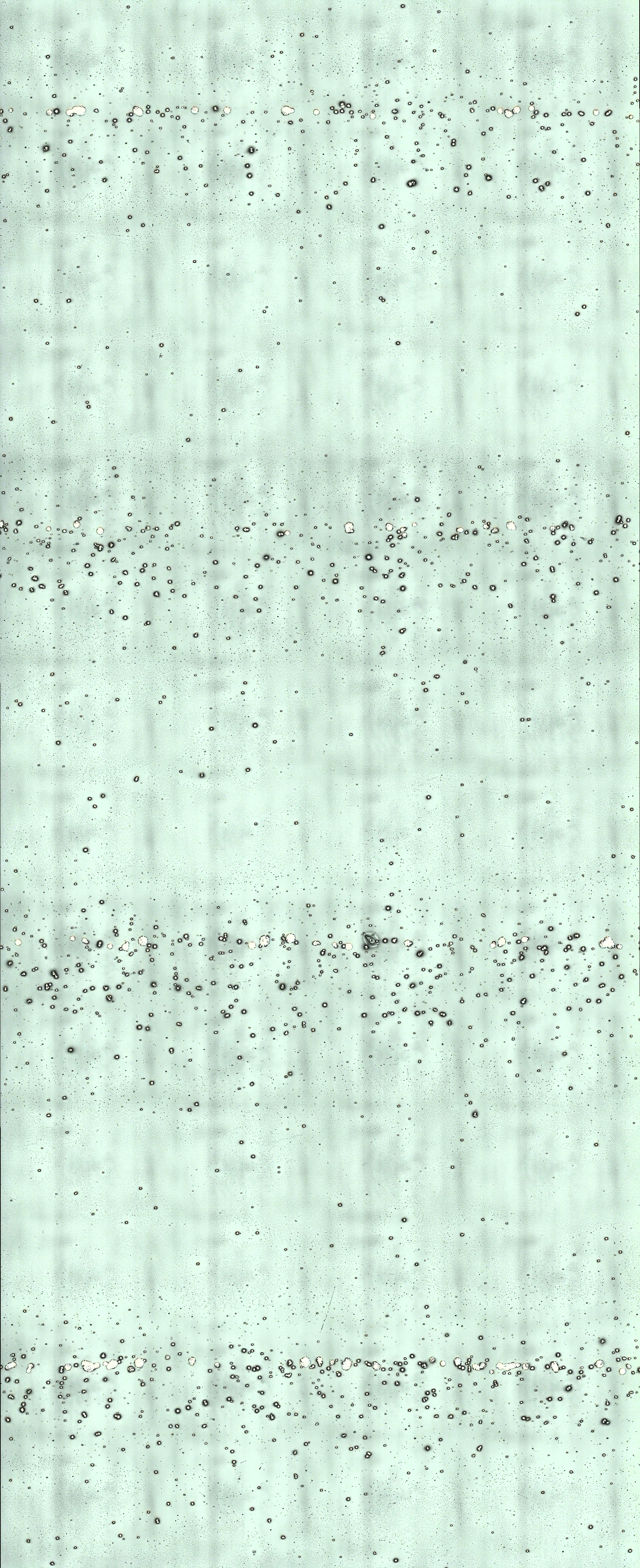

Supplement: Supplementary file 1 [file mmc1.zip › Optical_Image_24_to_38_mins.png]

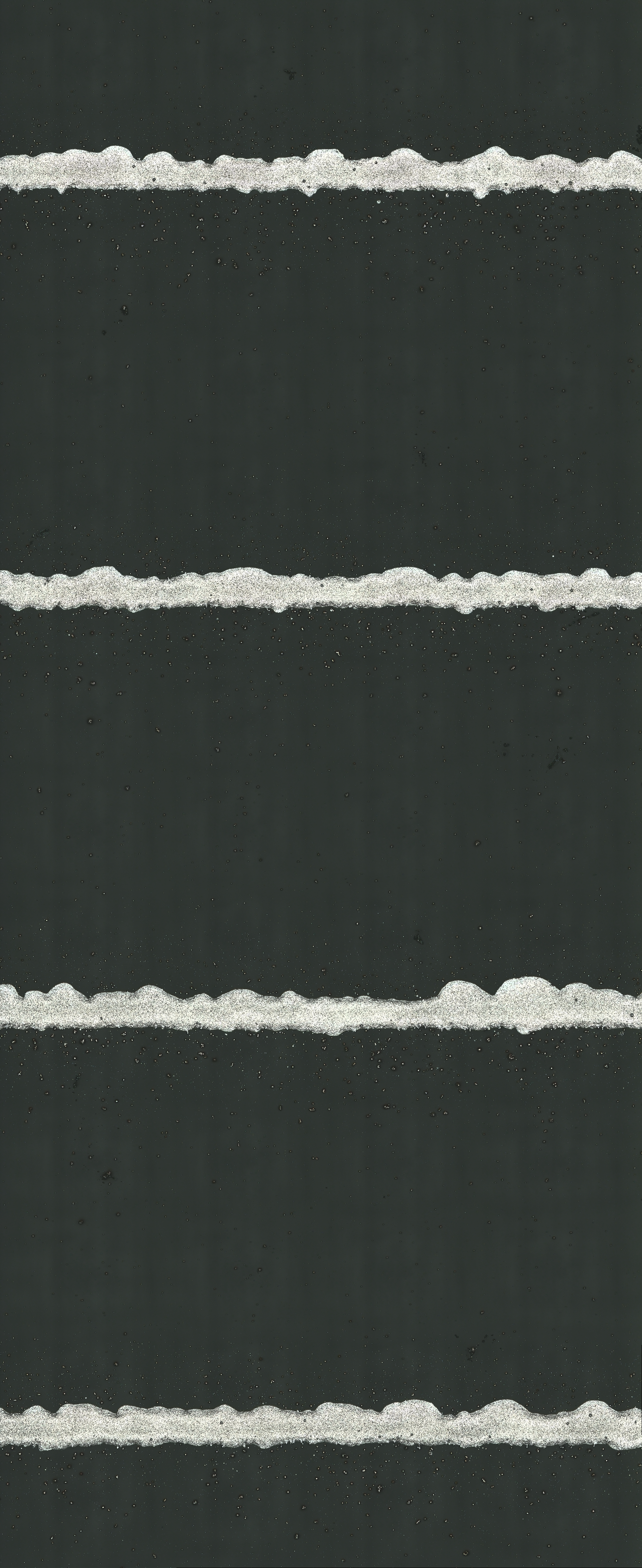

Supplement: Supplementary file 1 [file mmc1.zip › Optical_Image_293_to_307_mins.png]

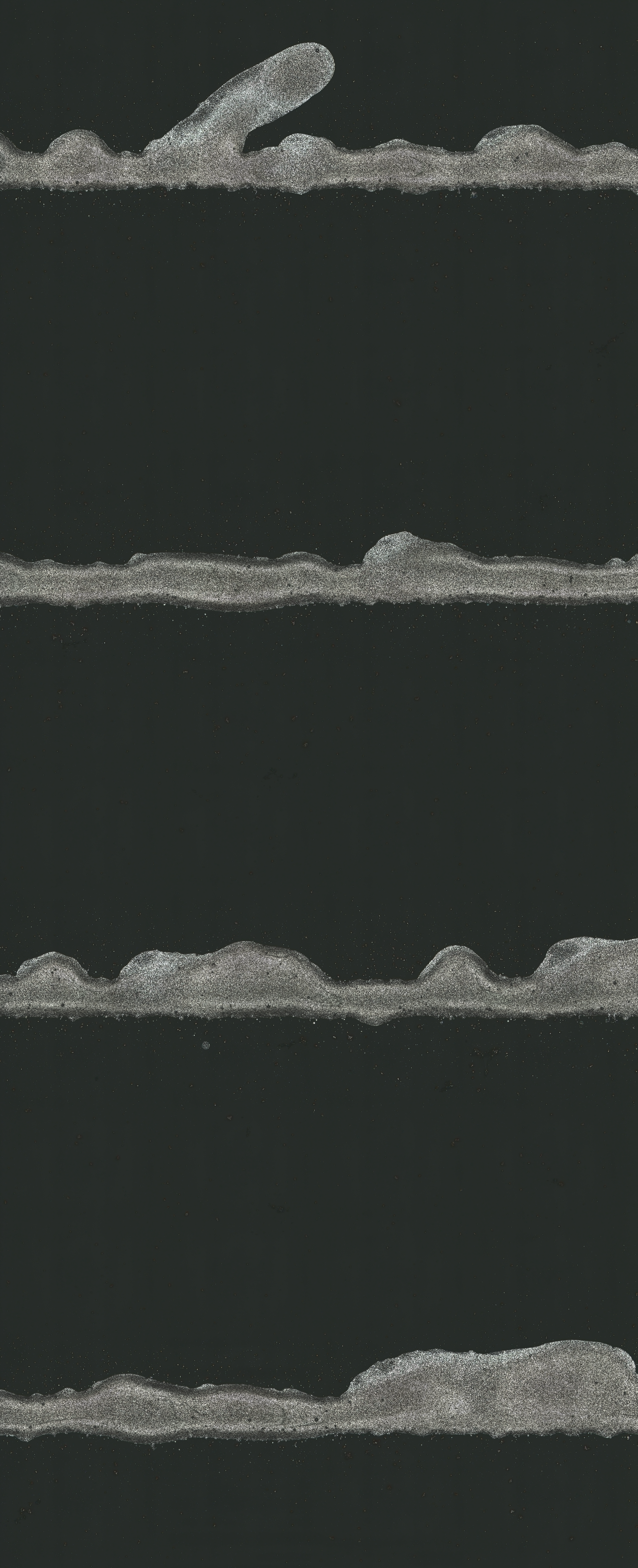

Supplement: Supplementary file 1 [file mmc1.zip › Optical_Image_341_to_355_mins.png]

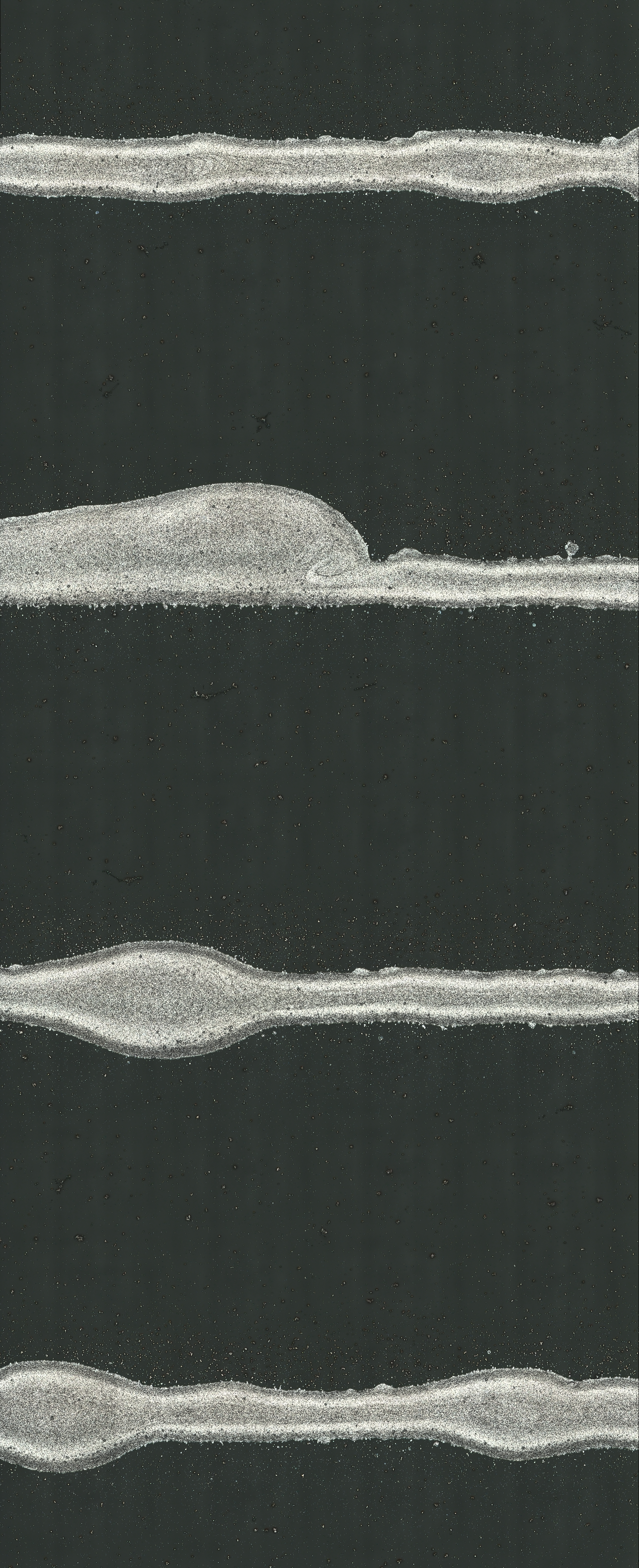

Supplement: Supplementary file 1 [file mmc1.zip › Optical_Image_389_to_403_mins.png]

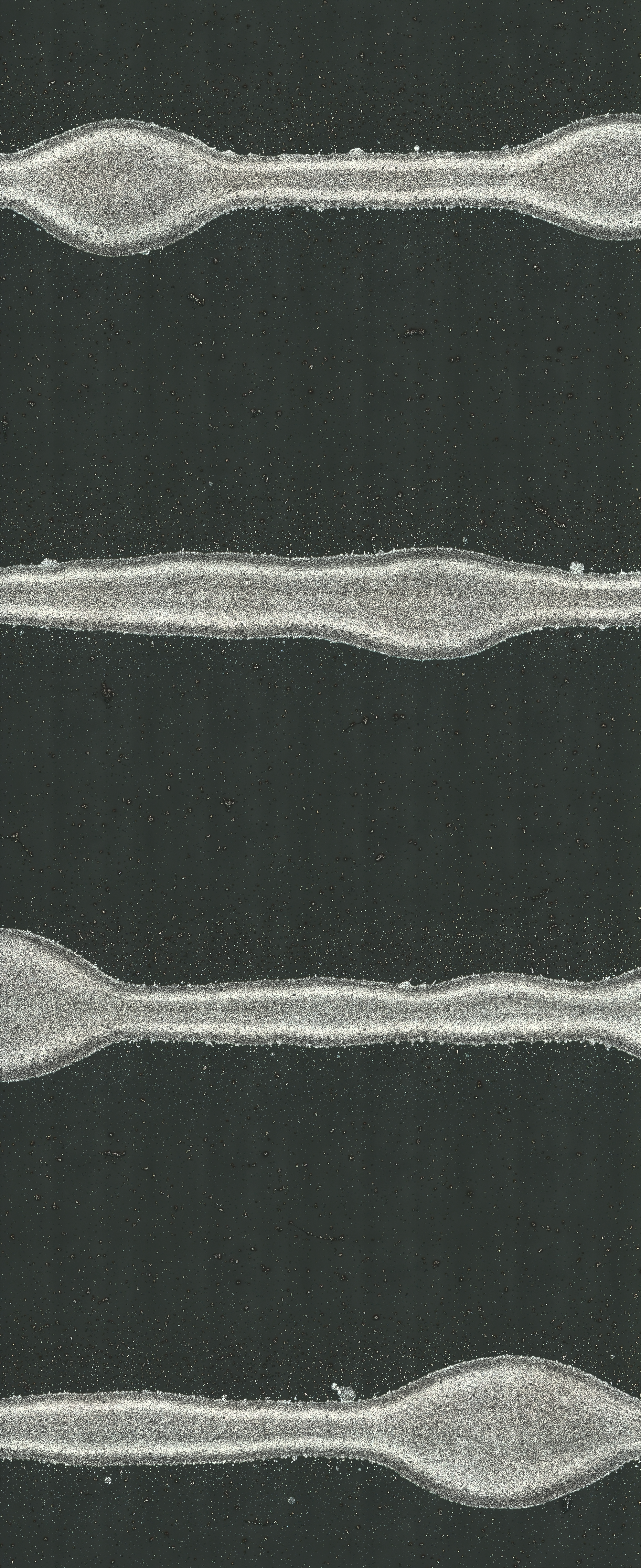

Supplement: Supplementary file 1 [file mmc1.zip › Optical_Image_437_to_451_mins.png]

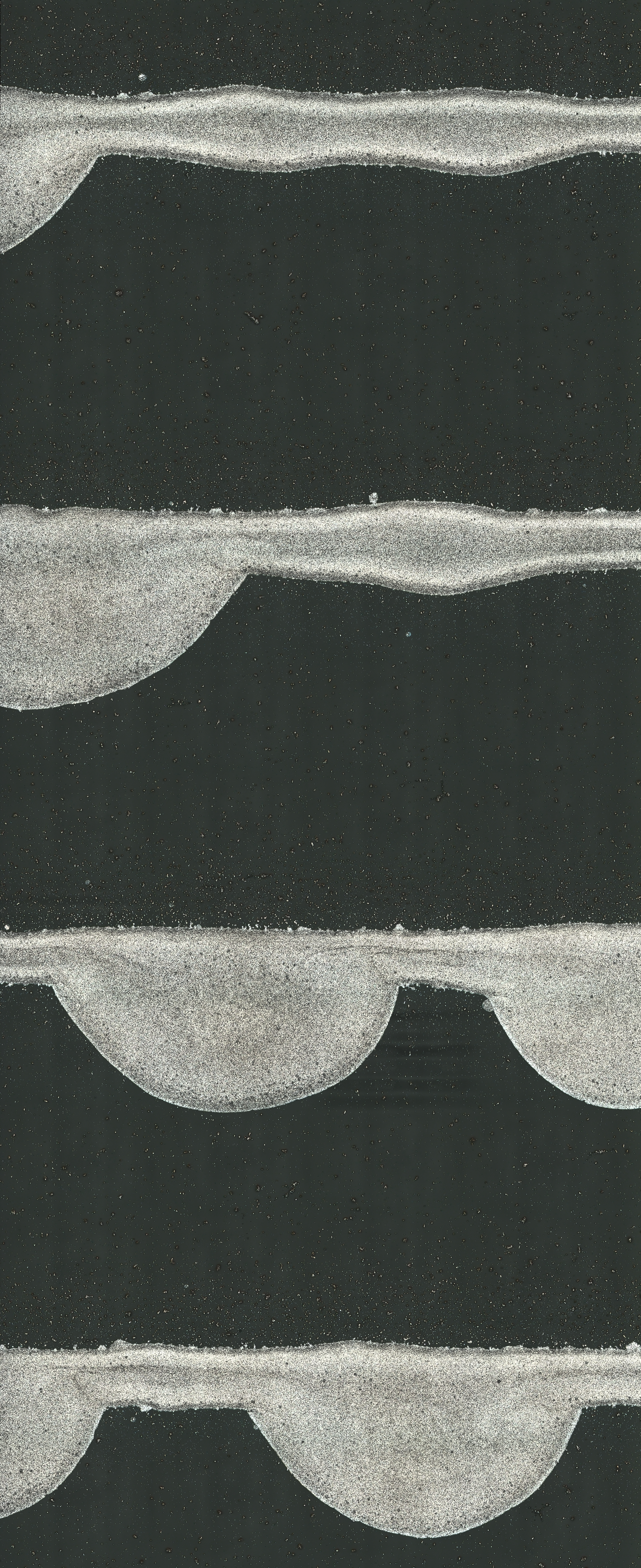

Supplement: Supplementary file 1 [file mmc1.zip › Optical_Image_485_to_499_mins.png]

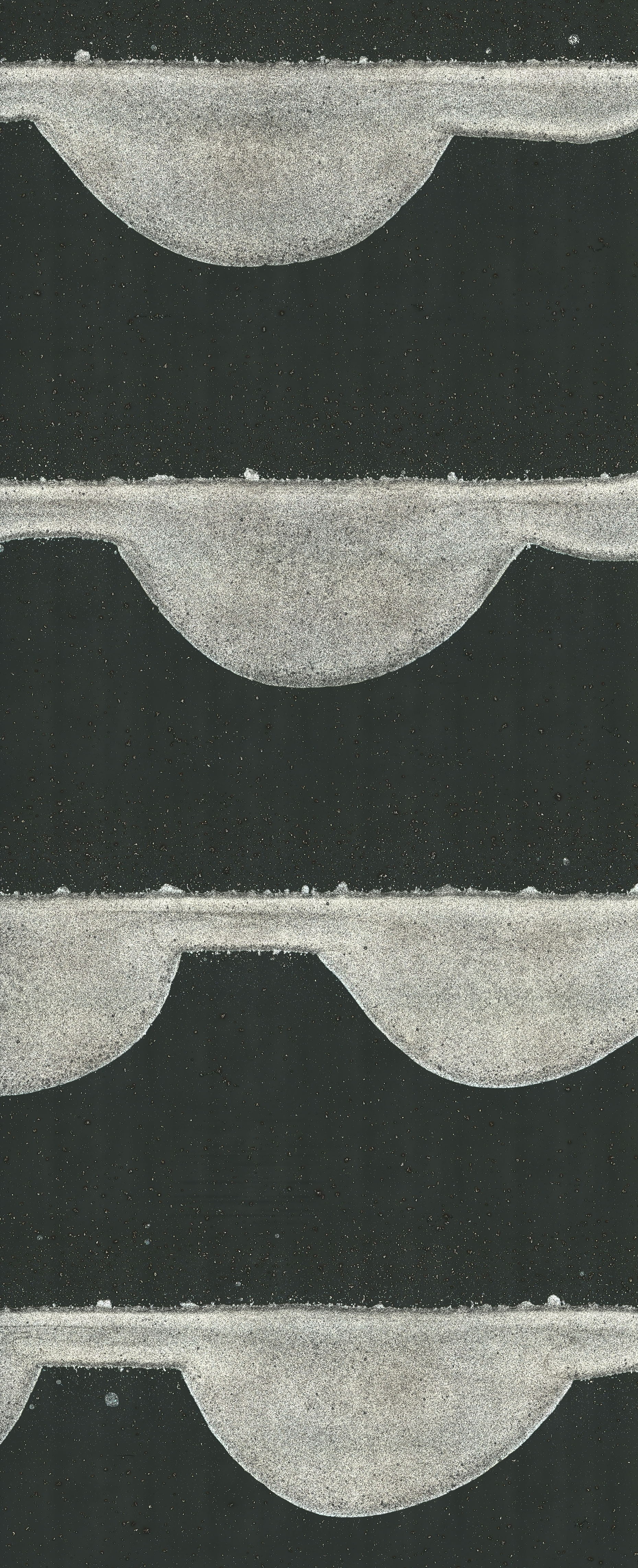

Supplement: Supplementary file 1 [file mmc1.zip › Optical_Image_533_to_547_mins.png]

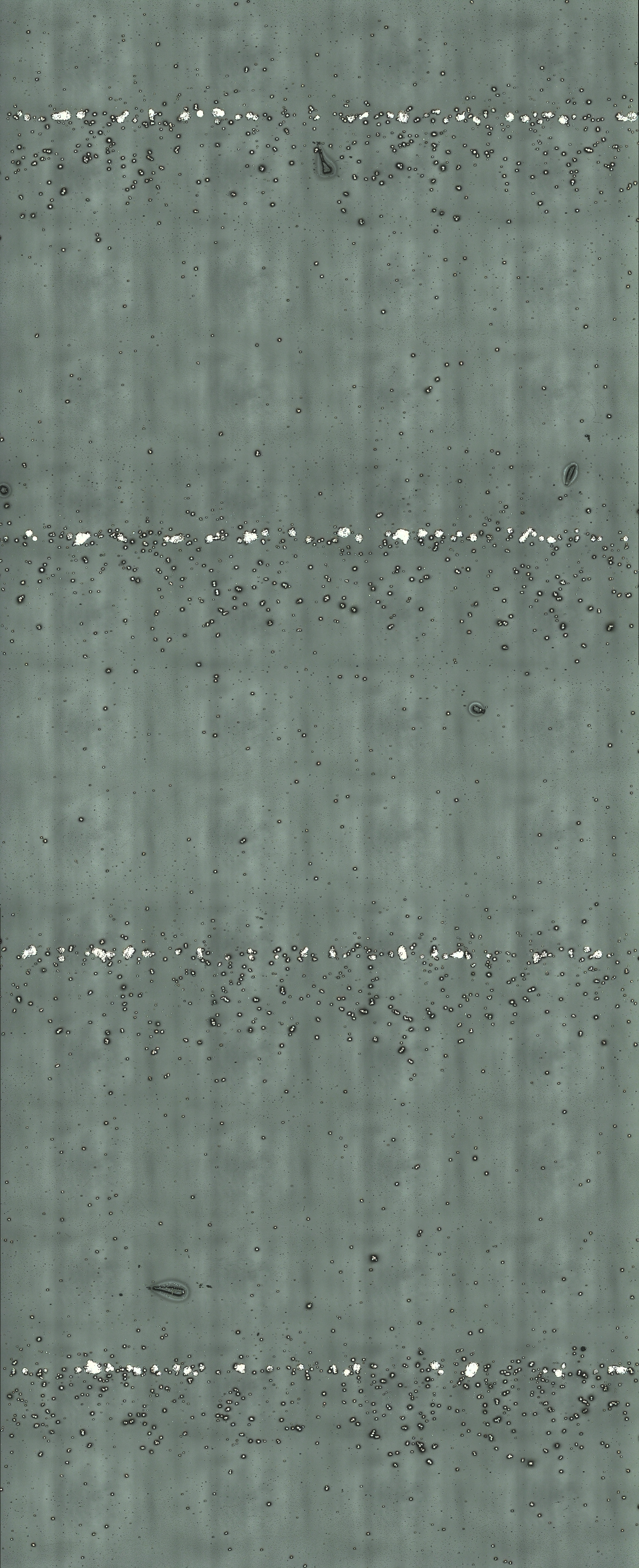

Supplement: Supplementary file 1 [file mmc1.zip › Optical_Image_53_to_67_mins.png]

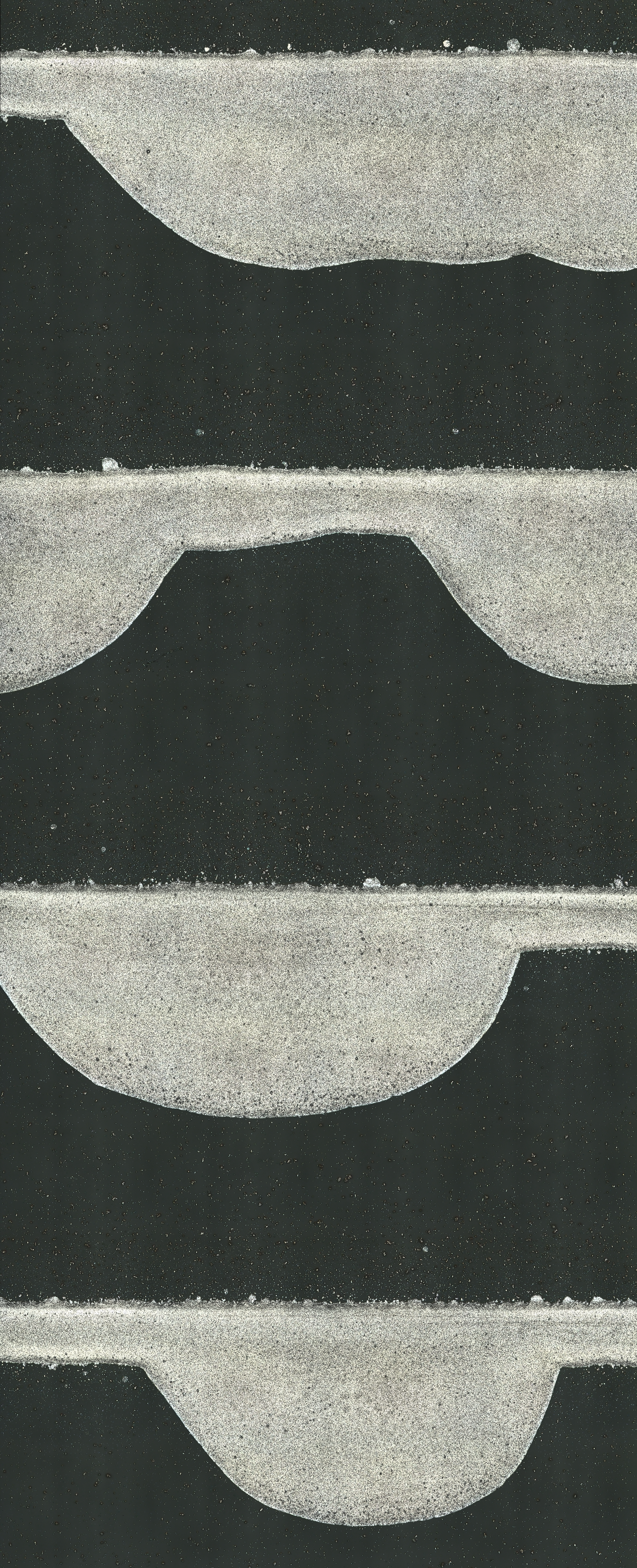

Supplement: Supplementary file 1 [file mmc1.zip › Optical_Image_581_to_595_mins.png]

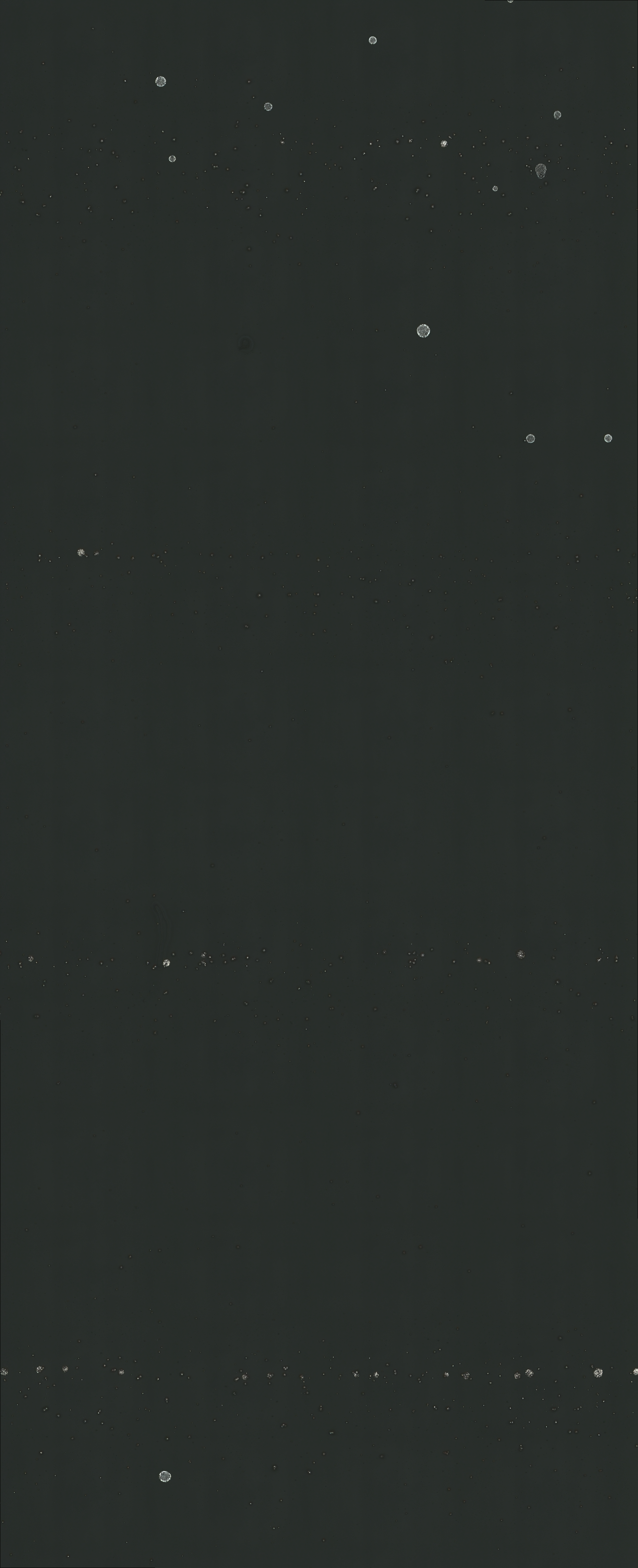

Supplement: Supplementary file 1 [file mmc1.zip › Optical_Image_5_to_19_mins.png]

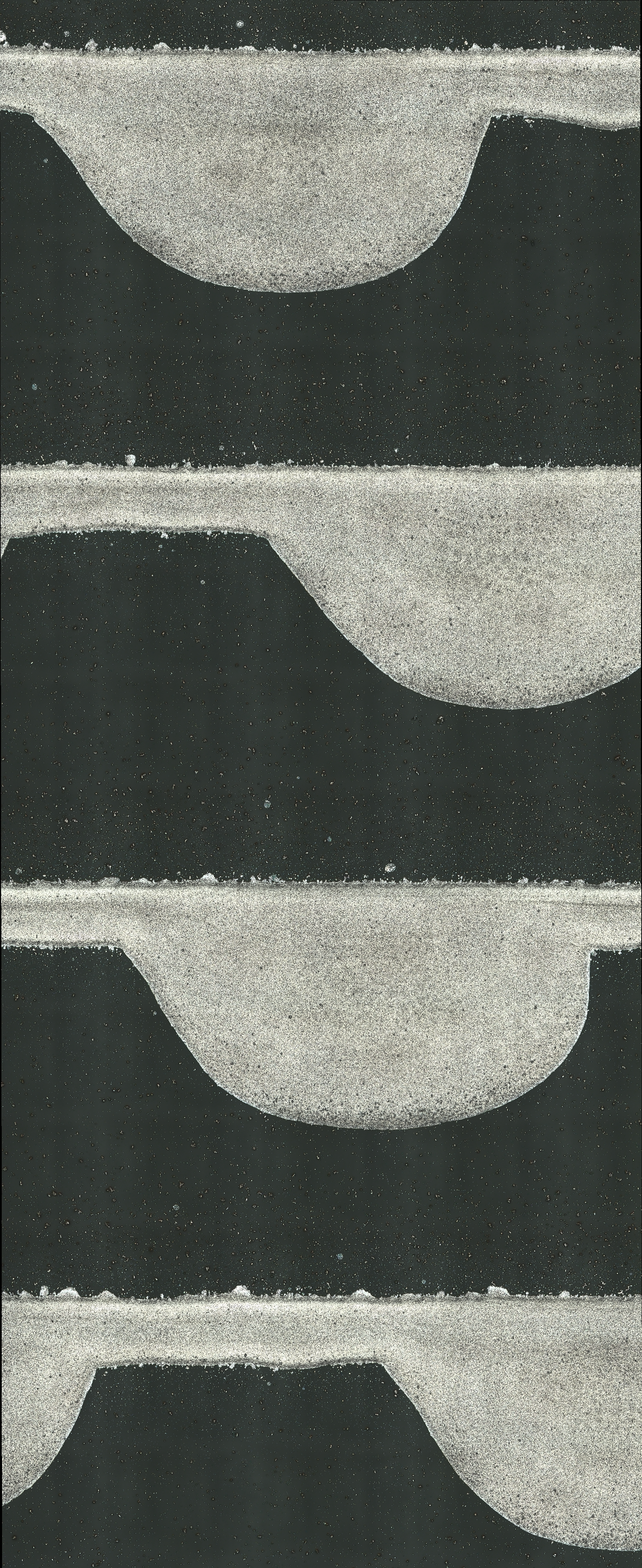

Supplement: Supplementary file 1 [file mmc1.zip › Optical_Image_629_to_643_mins.png]

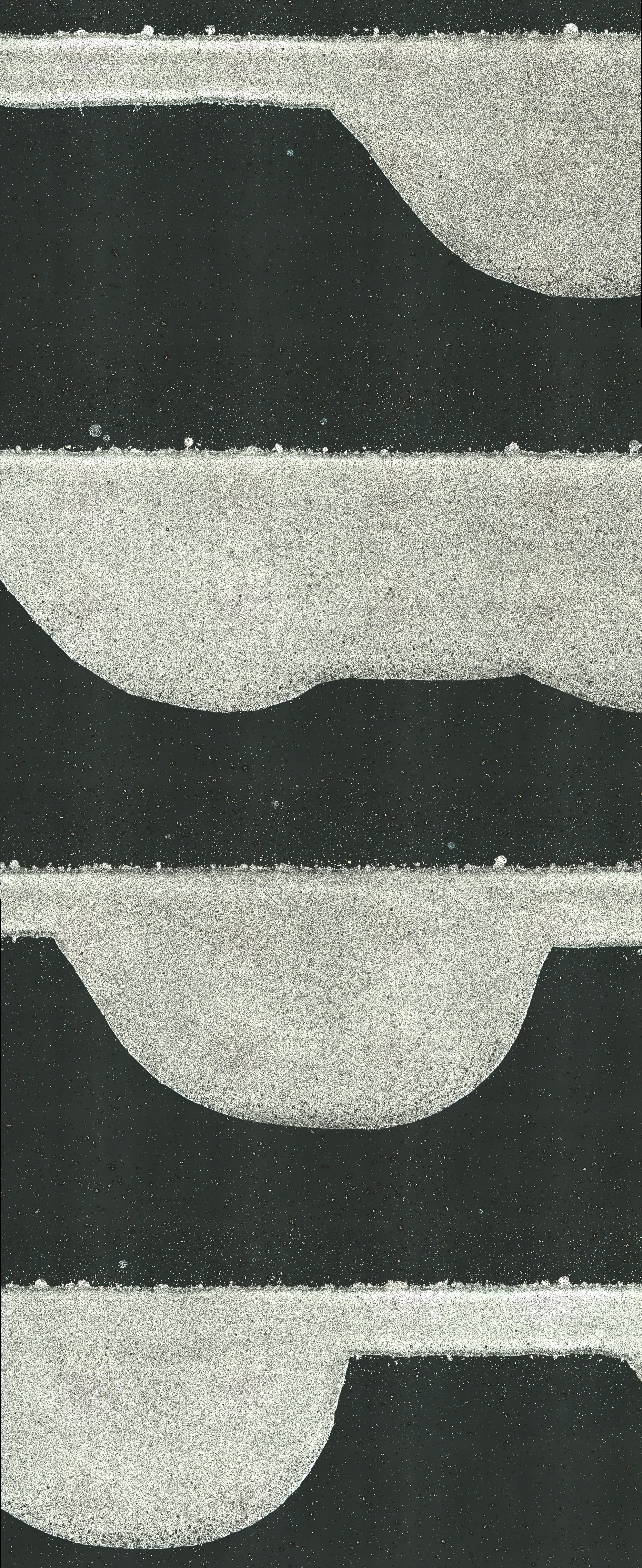

Supplement: Supplementary file 1 [file mmc1.zip › Optical_Image_677_to_691_mins.png]

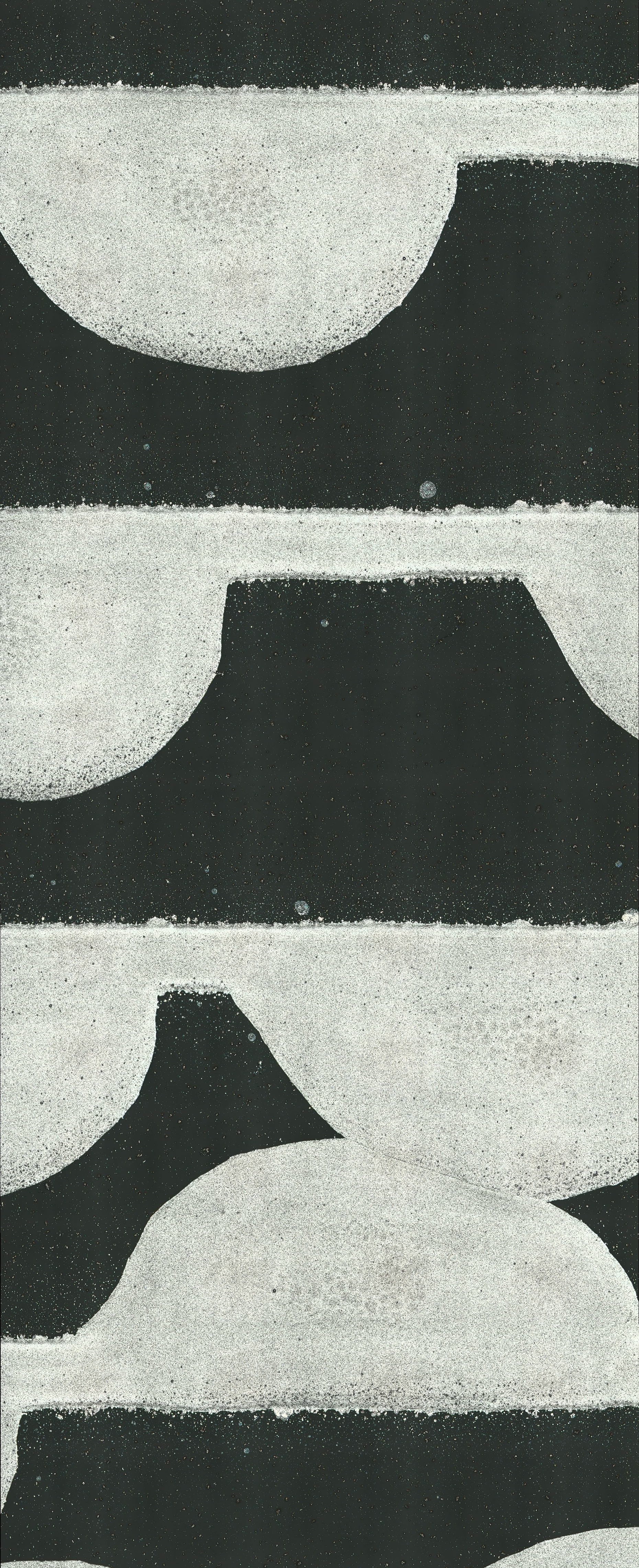

Supplement: Supplementary file 1 [file mmc1.zip › Optical_Image_725_to_739_mins.png]

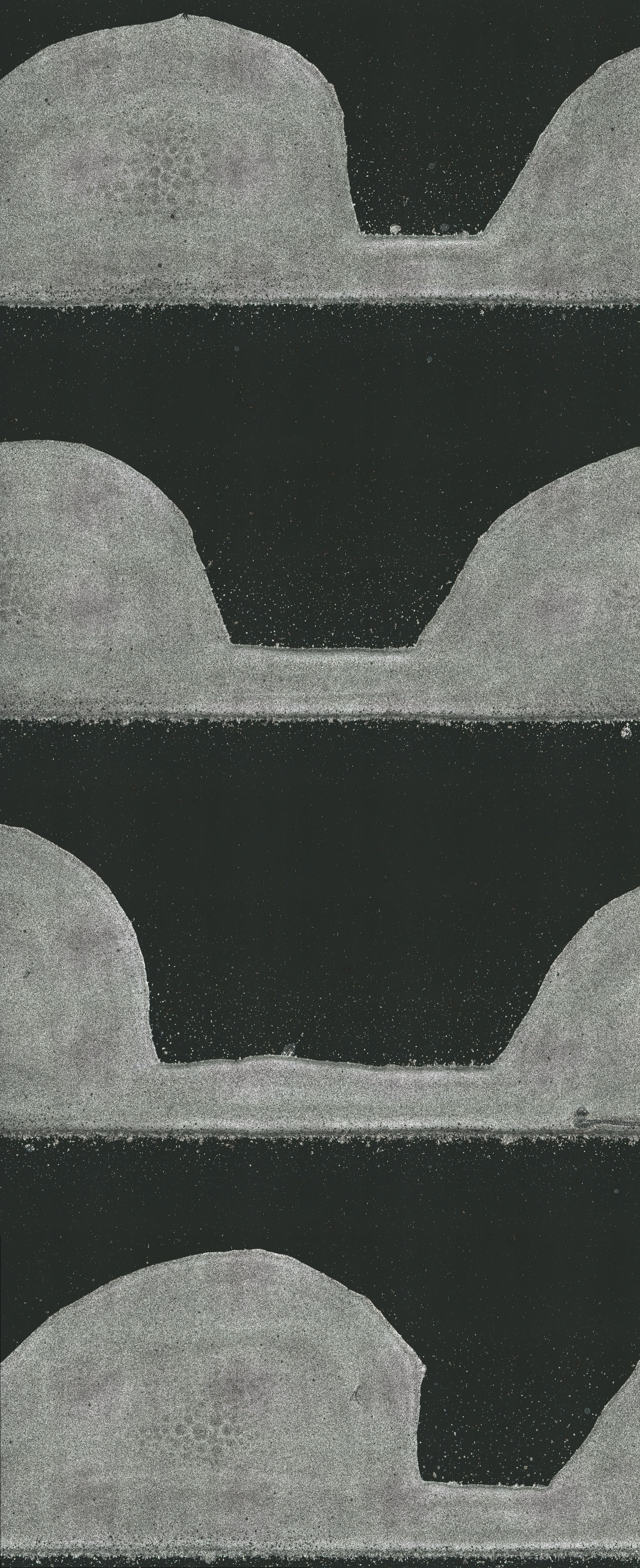

Supplement: Supplementary file 1 [file mmc1.zip › Optical_Image_773_to_787_mins.png]
